# A Case With Twists and Turns: A Report of Vertebrobasilar Dolichoectasia

**DOI:** 10.7759/cureus.61889

**Published:** 2024-06-07

**Authors:** Mary Therese Thomas, Jamie Campbell

**Affiliations:** 1 Internal Medicine, Grand Strand Medical Center, Myrtle Beach, USA

**Keywords:** vertebrobasilar junction, neuroradiology, vascular ectasia, microvascular decompression surgery, trigeminal nerve decompression, brain anatomy, neurology and neurosurgery, neurovascular compression

## Abstract

Vertebrobasilar dolichoectasia (VBD) is a rare anatomical abnormality of the vertebral artery system, defined as irregular expansion, elongation, and tortuosity of vertebral arteries. Anomalies of the vertebrobasilar artery can have a wide variety of clinical presentations, ranging from simple headaches to debilitating strokes. We present the case of an atypical presentation of VBD which mimicked trigeminal neuralgia by compressing the trigeminal nerve. There are currently no guidelines concerning the management of VBD, nor is there evidence of a definitive cure. This case invoked discussions among the medical team as to whether management should be medically or surgically focused, as well as long-term outcomes for patients with VBD. The superiority of medical versus surgical treatment of this issue is still a debated topic. This patient trialed medical management with dexamethasone and carbamazepine with no improvement in symptoms. He then underwent surgical gamma knife treatment but even this invasive measure was unsuccessful at relieving his symptoms. We hope that by presenting this case, we can display how the therapies available for VBD are limited and often unsuccessful in relieving the disease burden in patients with VBD.

## Introduction

Vertebrobasilar dolichoectasia (VBD) is an extremely rare anatomical abnormality of the vertebral artery system composed of the bilateral vertebral arteries and an unpaired basilar artery [1]. The basilar artery is the most common anomalous artery [2]. The incidence of symptomatic dolichoectasia is less than 0.05%; however, in those undergoing routine magnetic resonance imaging (MRI)/magnetic resonance angiography (MRA), the asymptomatic VBD incidence rate is 1.3% [3,4]. Ectasia of the basilar artery is defined as a diameter greater than 4.5 mm, with the typical average size of the basilar artery being 3.17 mm at the level of the pons [5,6]. Dolichoectasia is diagnosed using Smoker’s criteria which requires both ectasia and elongation of one of the arteries of the vertebral system on imaging [7].

Given the variations in vascular anatomy, clinical presentations can be quite broad. The most frequently reported symptomatic presentation is cerebral ischemia [3]. The twisting of the arterial branches of the vertebrobasilar arteries due to dolichoectasia decreases blood flow and causes ischemic symptoms [3]. Other more common presentations include hemorrhage, hydrocephalus, headache, or cranial nerve compression [3]. If the vessel is dolichoectatic, the patient will likely present with symptoms involving multiple cranial nerves [8]. If the vessel is elongated but normal in diameter, the deficit will likely be limited to a single cranial nerve [8]. The most commonly affected cranial nerve is the facial nerve, followed by the trigeminal nerve [8].

The physiology of VBD is poorly understood and is likely multifactorial. VBD has been associated with congenital diseases such as Marfan’s syndrome or autosomal recessive polycystic kidney disease [9]. Other potential etiologies include infections such as syphilis or expression of abnormal matrix metalloproteinases. The most agreed-upon risk factors include male sex, age, history of smoking, hypertension, and previous stroke [3,10].

There are currently no guidelines concerning the management of VBD, nor is there evidence of a definitive cure. Medical management of symptoms is typically the first-line therapy; however, larger-sized vascular ectasia increases the risk of adverse outcomes such as cerebral hemorrhage [3]. If a patient fails traditional medical therapies, surgical options are explored. These include microvascular decompression (MVD) and gamma knife radiosurgery (GKRS) [11].

In this case, we describe an atypical presentation of VBD in a patient who failed medical management, subsequently had worsening symptoms, and required GKRS. Even with treatment with medical and surgical interventions, his symptoms continue to be refractory to treatment, and he lives with daily pain from his VBD.

## Case presentation

A 77-year-old male with a past medical history of Parkinson’s disease, essential hypertension, hyperlipidemia, atrial fibrillation, and a previous basilar cerebrovascular accident approximately 15 years prior presented after five days of left-sided headache, blurred vision, and difficulty swallowing. During the five-day period, he had a worsening headache which was unrelieved with over-the-counter abortive therapies. The patient’s inability to sleep the night before prompted his presentation. He described the headache as primarily left-sided, originating at the angle of his jaw and radiating across his entire face in the distribution of V1, V2, and V3. He had been unable to shave due to the pain and hypersensitivity. Additionally, he reported bilateral visual blurriness. The pain was described as intermittent and stabbing for a few seconds, followed by a persistent, residual dull pain. The pain did not worsen with chewing but worsened with movement, particularly with standing. He denied any recent head trauma, fever, pain, chills, constipation, diarrhea, chest pain, shortness of breath, sore throat, rhinorrhea, fevers, or cough. Additionally, the patient had received the shingles vaccine a month prior and had noted bumps in both armpits which wrapped around his back and were associated with a burning sensation.

On examination, the patient was a pleasant, appropriate-for-age male in a moderate amount of distress. He was alert and oriented to person, place, time, and situation. The heart, lung, and abdominal examinations were normal. There was slightly diminished strength on his left upper and lower extremities with an intermittent tremor/jerking of the right upper extremity and some neck twitching to the right, both of which were baseline, according to the patient. The patient’s pupils were symmetric and reactive, with movement intact. He had a symmetric smile with a globally intact sensation. The ear canals were clear, with translucent tympanic membranes and no air-fluid level. The left external pinna was tender to manipulation. An area of bilateral axillary erythema with some scaling and healing vesicles in a non-dermatomal distribution were present, and there was erythema to the left eyelid with normal conjunctiva bilaterally.

**Figure 1:**
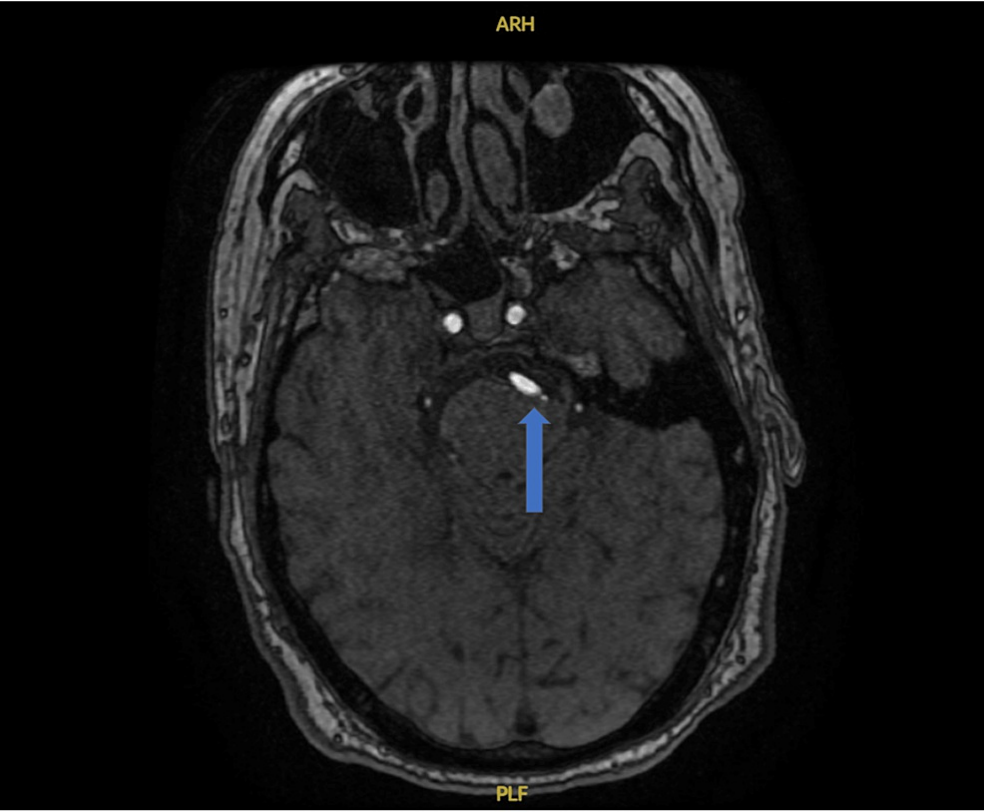
Dominant right vertebral artery crossing the midline to the confluence with the vertebral artery, with a return midline course of the basilar artery along the left ventral aspect of the brainstem resulting in mild compressive deformity.

**Figure 2:**
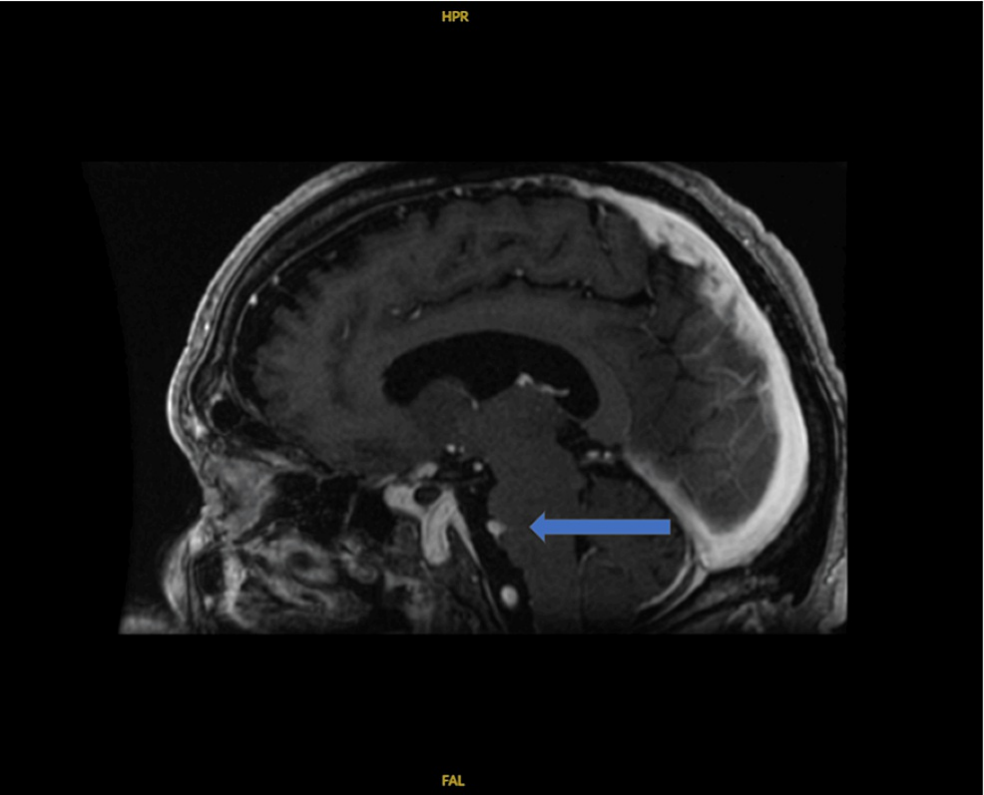
Sagittal view of mild compressive deformity caused by the right vertebral artery.

**Figure 3:**
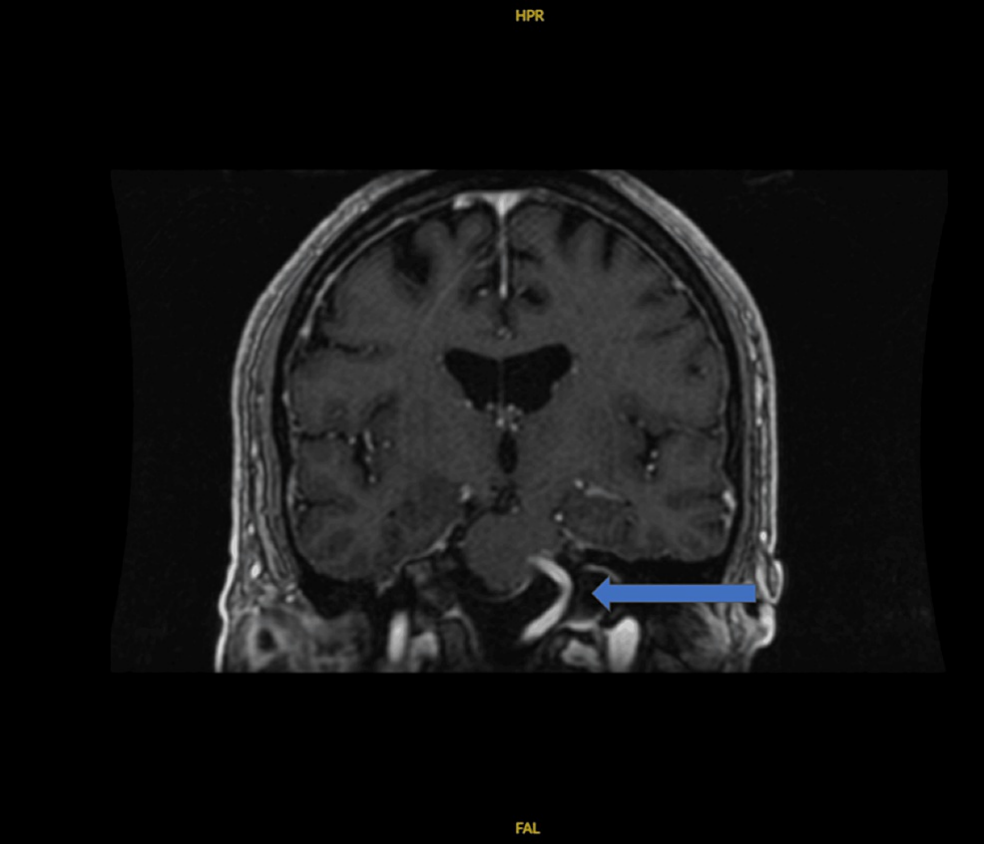
Coronal view of mild compressive deformity caused by the right vertebral artery.

**Figure 4:**
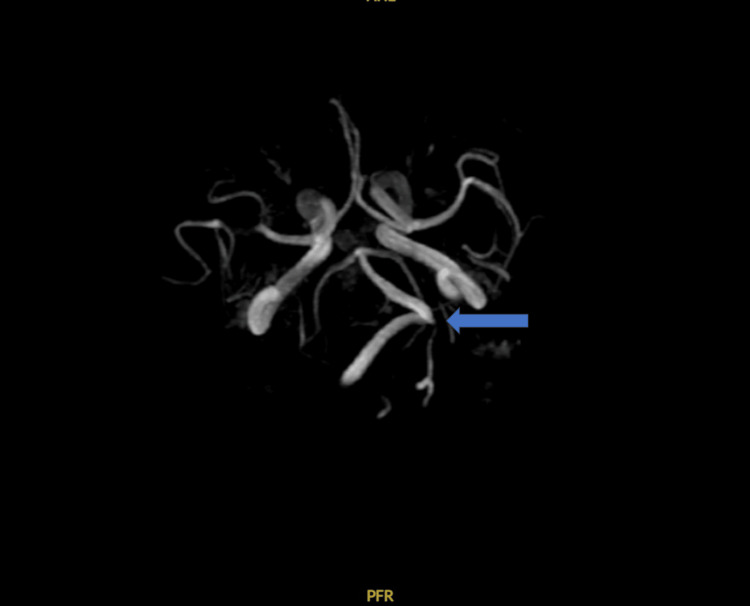
Clear dolichoectasia of the vertebral artery with a loop directed to the left toward the neurovascular complexes.

The patient’s initial workup was notable for a platelet count of 83 µL, C-reactive protein <0.5 mg/dL, and erythrocyte sedimentation of 1 mm/hour. Presenting vital signs were within normal limits. CT of the head without contrast was negative for intracranial findings but scattered mucosal inflammatory disease suggestive of possible developing acute sinusitis was noted. MRI and MRA with contrast revealed VBD with mild compressive deformity of the left ventral brainstem, with suspected neurovascular compression of the cisternal segment of the left trigeminal nerve (Figures 1-4).

The patient’s headache and facial sensitivity/pain proved difficult to control. A multimodal pain regimen was used including ketorolac, tramadol, gabapentin, and morphine with minimal successful pain control. Both Neurology and Neurosurgery were consulted concerning the MRI findings. Both teams recommended an attempt at medical management and noted no acute or emergent surgical intervention. This medical plan included treatment of the acute sinusitis with amoxicillin/clavulanic acid 500-125 mg every eight hours for five days and a limited trial of dexamethasone 10 mg once followed by 2 mg tablets every eight hours for one week with outpatient follow-up. For pain control, Neurology recommended starting carbamazepine 200 mg twice a day and tramadol 50 mg every six hours, as needed.

After discharge, the patient completed his course of antibiotic therapy and continued using the prescribed scheduled multimodal pain regimens. He continued to have severe, unrelenting pain. He was seen at an outside university hospital and underwent GKRS approximately three months after the initial discharge. While he had about a 30% reduction in his pain immediately postoperatively, a year post-surgery, the pain had returned to the same severity as at the initial presentation. During that year, he was also diagnosed with an enlarging thoracic aneurysm and was planning to undergo surgical repair. At the time of writing this paper, he continues to search for alternative therapies to help relieve his daily pain.

## Discussion

The first-line medicines used to control symptoms of neurological pain from VBD include antiseizure medications such as phenytoin and carbamazepine, with the latter being the first-line medication for trigeminal neuralgia [12]. If symptoms are unable to be controlled with medical management, then surgical options are considered. The most popularized procedures are MVD and GKRS.

There have been many reviews regarding the efficacy of MVD. It is a technique created in the 1960s that places a synthetic padding between the vasculature and the nerve [13]. Overall, there has been much success with this technique but the technicality of the procedure itself requires highly specialized training and, many times, does not provide satisfactory long-term outcomes [14,15]. Complications that occur are serious such as intraoperative need for conversion to craniotomy and postoperative cerebral infarction and subsequent edema [16]. Goldenberg-Cohen et al. did not recommend MVD for cases with single nerve involvement as risks outweigh the postoperative benefits and outcomes [10].

In the more elderly or frail patients who may not be able to tolerate MVD, other surgeons may turn to the less invasive GKRS as an alternative therapy. GKRS aims to focus small radiation beams at the targeted vessel to decrease the overall size and therefore symptom burden after relief of nerve compression [13]. However, long-term outcomes for maintenance control are very poor and even inferior to MVD [17].

This leaves the field open for innovative surgical techniques to provide superior outcomes to these known interventions. Electrical neurocoagulation, cutting of the tentorium, endovascular stenting, and scarring of the dura to reposition the basilar artery are techniques that are being explored as alternative treatments to MVD and GKRS [18-21]. Some claim to even have fewer complications, better long-term outcomes, and require less procedure technicality.

Currently, regardless of intervention, the five-year mortality for patients with VBD is 36.3% [14]. Unfortunately, suicide can become seemingly more reasonable for those with untreated symptoms of VBD than living with the pain [21]. The need for further investigation to better understand the disease, risk factors, consequences, and treatment of VBD is imperative for these patients.

## Conclusions

On the initial presentation of this case, the differential diagnosis included trigeminal neuralgia or giant cell arteritis. MRI/MRA revealed the extremely rare diagnosis of VBD causing trigeminal pain. After unsuccessful medical management with carbamazepine, dexamethasone, and multimodal pain regimens, the patient underwent GKRS. While initially successful, the long-term treatment outcome was poor and he had a return of symptoms and pain within one year. Other popularized surgical interventions pose a more involved and complex surgical intervention than GKRS, so the patient continues to search for other treatment modalities to treat his persistent pain. Understanding the risk factors, physiology, and pathology and exploring curative treatments for VBD is important and necessary to prolong life in these individuals.
